# Concomitant coronary and pulmonary embolism associated with patent foramen ovale: A case report

**DOI:** 10.1097/MD.0000000000009480

**Published:** 2017-12-29

**Authors:** Zhongxiu Chen, Chen Li, Yajiao Li, Hong Tang, Li Rao, Mian Wang

**Affiliations:** Department of Cardiology, West China Hospital of Sichuan University, Chengdu, Sichuan, China.

**Keywords:** paradoxical embolism, patent foramen ovale, transesophageal contrast echocardiography

## Abstract

**Rationale:**

The differential diagnosis of acute chest pain is very important, and can sometimes be challenging. Related diseases share a number of risk factors, and occasionally, 1 condition causes another disease to develop.

**Patient concerns:**

We described a 59-year-old man who presented to emergency department complaining of chest pain.

**Diagnoses:**

He was suffered acute myocardial infarction (MI) and pulmonary embolism (PE) simultaneously.

**Interventions:**

Dual antiplatelet therapy, statin, and low molecular weight heparin were administrated during his stay. The searches for cancers, autoimmune diseases, and hematologic diseases were unremarkable, ruling out a hypercoagulable state. Subsequent ultrasound scan revealed a thrombus in a vein of the lower left extremity. Thus, paradoxical embolism was highly suspected.

**Outcomes:**

Paradoxical embolism is a rare cause of acute MI, which may have occurred in our patient. This was evidenced by a previously unrecognized patent foramen ovale (PFO) with a right-to-left atrial shunt detected using contrast transesophageal echocardiography.

**Lessons:**

Acute MI complicated with PE is not common in the clinical setting. The fatal condition is difficult to diagnose because of the similar symptoms and confusing causes. Paradoxical embolism can cause this phenomenon, and physicians should be highly vigilant in the search for a PFO in cases of paradoxical embolism.

## Abstract 2

Supplemental Digital Content is available in the text

## 1 Introduction

Diseases related to acute chest pain are common and life-threatening conditions. These may present with similar symptoms, leading to misdiagnosis and prolonged therapy. Acute myocardial infarction (MI) and pulmonary embolism (PE) are 2 common causes of acute chest pain. However, the concomitant occurrence of these 2 life-threatening conditions is rare. This does not necessarily violate the principle of parsimony, because the simultaneous onset of MI and PE is usually related to a single underlying disease, for example, hypercoagulable state or paradoxical embolism.

## 2 Case presentation

A 59-year-old man was brought to the emergency department (ED) complaining of chest pain radiating to the throat and shortness of breath for 5 h. He had a previous history of hypertension, which was not well-controlled, and a 30 pack-year history of smoking. His heart rate was 84/min, respiratory rate was 24/min, oxygen saturation was 85% on room air, blood pressure was 114/85 mm Hg, and body temperature was 36.3°C in the ED. Physical examination revealed cyanotic lips, and clammy and cool extremities.

**Figure 1:**
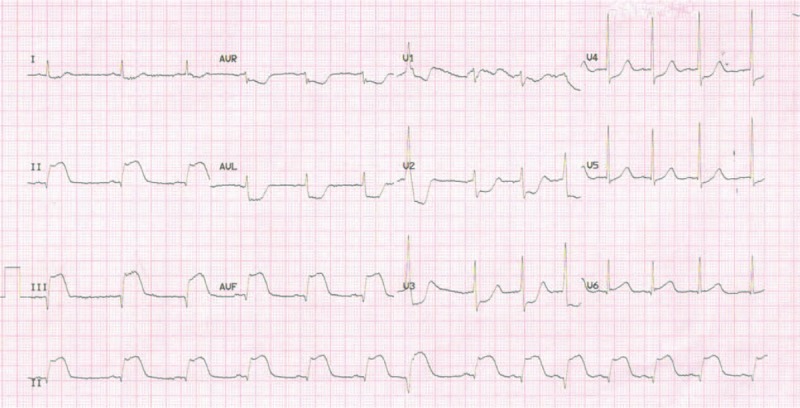
Twelve-lead electrocardiogram showing ST elevations in inferior leads.

**Figure 2:**
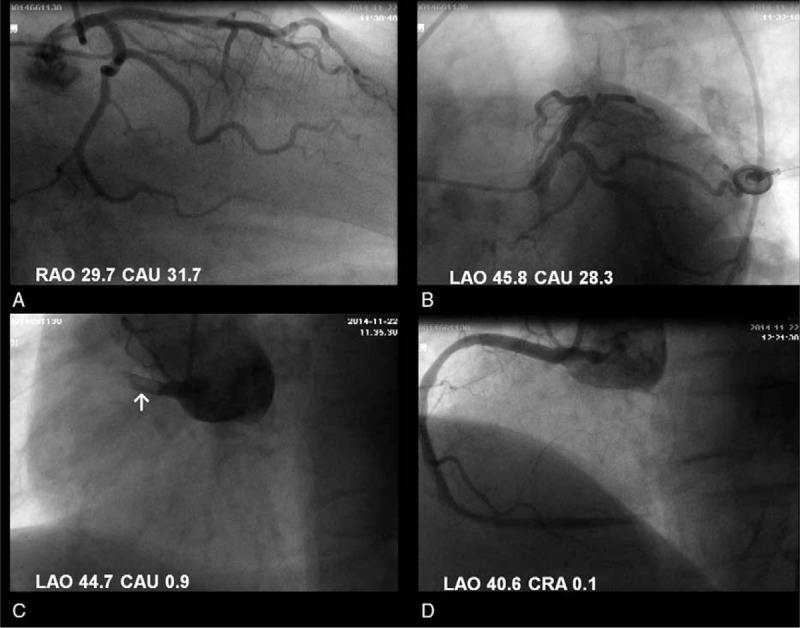
Coronary angiogram showing thrombus in the right coronary artery (RCA). (A and B) No significant stenosis was detected in the left coronary artery. (C) A total occlusion at the beginning of the RCA (white arrow). (D) After transcatheter thrombus aspiration, the blood flow to the RCA was fully restored and no ulcerated atheromatous plaque was observed.

**Figure 3:**
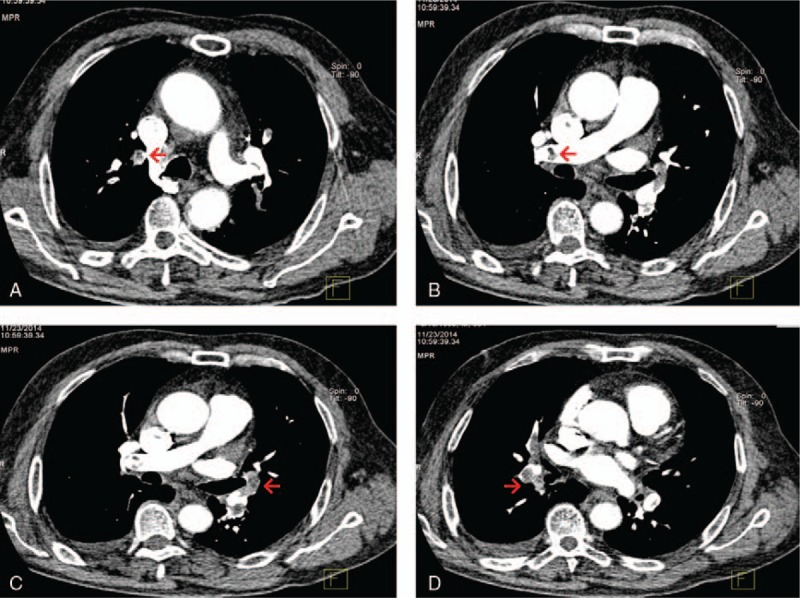
Computed tomography image showing pulmonary embolism (red arrows). A-D, showing PE in different scanning levels.

A 12-lead electrocardiogram performed in the ED revealed ST elevations in the inferior leads (Fig. 1). Cardiac troponin T and creatine kinase-MB were significantly elevated (258.4 ng/L and 29.92 ng/mL, respectively). Thus, inferior wall ST-segment elevation MI was highly suspected. A subsequent emergent coronary angiography showing total occlusion at the beginning of the right coronary artery confirmed the diagnosis of acute MI (Fig. 2). After trans-catheter thrombus aspiration, the blood flow to the right coronary artery was fully restored. No ulcerated atheromatous plaque was observed, indicating the possibility of thromboembolism. As a result, stent implantation was not performed. The patient was transferred to the cardiac intensive care unit afterward. His chest pain was relieved immediately and his vital signs were stable, except for persistent hypoxemia even after oxygen inhalation. Bedside blood gas analysis showed the arterial blood oxygen partial pressure to be 47.7 mm Hg. His hypoxemia was not explained by the MI, as no signs of heart failure or shock were observed. To further search for the potential causes of the hypoxemia, enhanced computed tomography scan of the pulmonary artery was carried out, which revealed extensive bilateral PE (Fig. 3).

**Figure 4:**
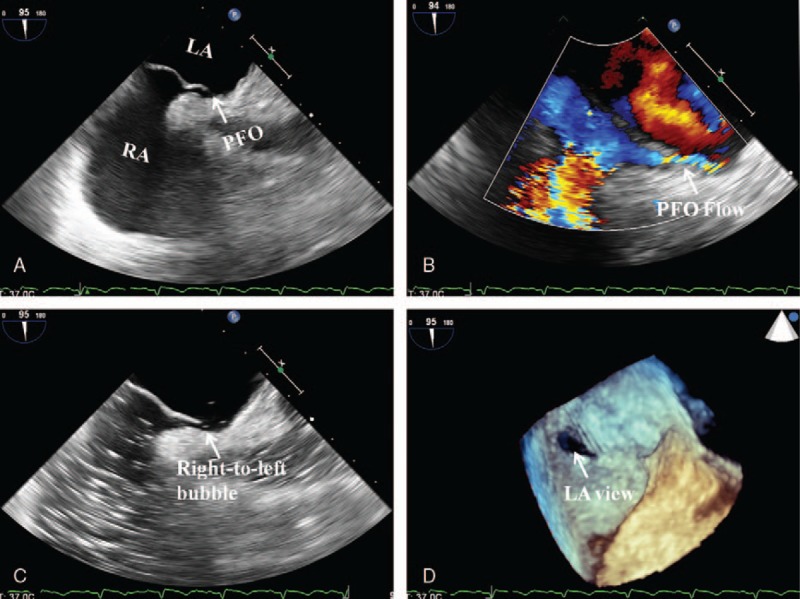
Transesophageal echocardiography (TEE) confirming the PFO (arrow). (A) The 2-dimensional TEE in the biatrial view detected a separation between the primum and secundum atrial septum. (B) Color Doppler demonstrated bilateral but mainly right-to-left flow by decreasing the color gain and wall filtration. (C) Contrast TEE revealed right-to-left shunt after the contrast agent (6 mL of 1% injection vitamin B6 and equal volume of 5% sodium bicarbonate solution) was administered through the dorsal vein of right hand. (D) Real-time 3-dimensional TEE confirmed the PFO. LA = left atrium, PFO = patent foramen ovale; RA = right atrium.

Because the patient suffered thrombotic events in the systemic and pulmonary circulations simultaneously, paradoxical embolism or a hypercoagulable state was highly suspected. The searches for cancers, autoimmune diseases, and hematologic diseases were unremarkable (Supplemental Tables 1–3), ruling out a hypercoagulable state. Ultrasound scan of the lower extremities revealed a thrombus in a vein of the left extremity. However, bedside transthoracic echocardiography failed to identify any obvious defects or shunts (Supplemental Fig. 1). Although the transthoracic echocardiography showed no evidence of any defect, we still thought that paradoxical embolism via a patent foramen ovale (PFO) was the best explanation for the phenomenon. After the patient was stabilized, transesophageal contrast echocardiography was performed, which revealed bilateral (mainly right-to-left) shunting through the PFO (Fig. 4). Thus, the patient was diagnosed with PE and inferior MI secondary to paradoxical embolism. He received dual antiplatelet therapy, statin, and low molecular weight heparin during his stay. Ten days later, he was discharged in a stable condition with dual antiplatelet therapy, statin, and oral vitamin K antagonist. At follow-up 6 months after the event, the patient was free from symptoms.

The case protocol was approved by the Ethics Committee of the West China Hospital of Sichuan University (Sichuan, China). Informed consent was obtained from the patient for publication of this case.

## 3 Discussion

Although PE is not rare in patients with severe cardiovascular diseases, misdiagnosis and prolonged therapy of this fatal condition sometimes occurs because of the similar symptomatology and confusing causes.^[1]^ In a large autopsy study, PE was found in 24.4% of patients who died of cardiovascular diseases, of which 82% were not recognized before death.^[2]^ This stunningly high misdiagnosis rate might be because of the many similar findings of acute PE and acute coronary syndrome or heart failure. Physicians should be vigilant of signs like unexplained dyspnea, elevated D-dimer, or right ventricular distention.^[3]^ In our case, the inferior wall infarction could not explain the respiratory distress and hypoxia, which is mainly observed in large anterior infarctions resulting in acute heart failure or with acute mechanical complications.

PE is common in patients with severe heart diseases, usually because of long-term immobilization, but simultaneous occurrence of PE and MI is relatively rare. Most of these cases are related to paradoxical embolism through a PFO. One reason for this is that the prevalence of PFO is about 27%^[4,5]^; that is, it is much more common than hypercoagulability. More importantly, transient right-to-left shunt caused by elevated pulmonary pressure in the presence of PE has been noticed decades ago.^[6]^ Although the Young Adult Myocardial Infarction and Ischemic Stroke study failed to show that the presence of a PFO increased the risk of paradoxical embolism in the general population,^[7]^ patients with acute PE nevertheless had a much higher risk of paradoxical embolism.^[8]^

The role of PFO closure in paradoxical embolism remains controversial. To date, no randomized controlled trials have shown clear net clinical benefit of PFO closure. The American Heart Association/American Stroke Association joint guidelines suggested that PFO closure for patients with cryptogenic ischemic stroke should be individually tailored, depending on the risk of recurrent deep vein thrombosis.^[9]^ As patients who received adequate anticoagulant treatment had very low recurrence rates of PE,^[10]^ PFO closure might not be necessary for our patient.

## Supplementary Material

Supplemental Digital Content
