# Supplementary material for: Concomitant coronary and pulmonary embolism associated with patent foramen ovale: A case report
Source: Medicine (Baltimore). 2017 Dec 29;96(52):e9480. doi: 10.1097/MD.0000000000009480 (PMC6392996; doi:10.1097/MD.0000000000009480)
Supplement: Supplemental Digital Content [file medi-96-e9480-s001.doc]

**Supplemental figure 1. No defects was identified in atrial septa through transthoracic echocardiography (TTE).**

**A.** No defects was identified in atrial septa in the subcostal biatrial plane of two-dimensional TTE. **B.** No definite flow across were showed in color Doppler. RA, right atrium; LA, left atrium.


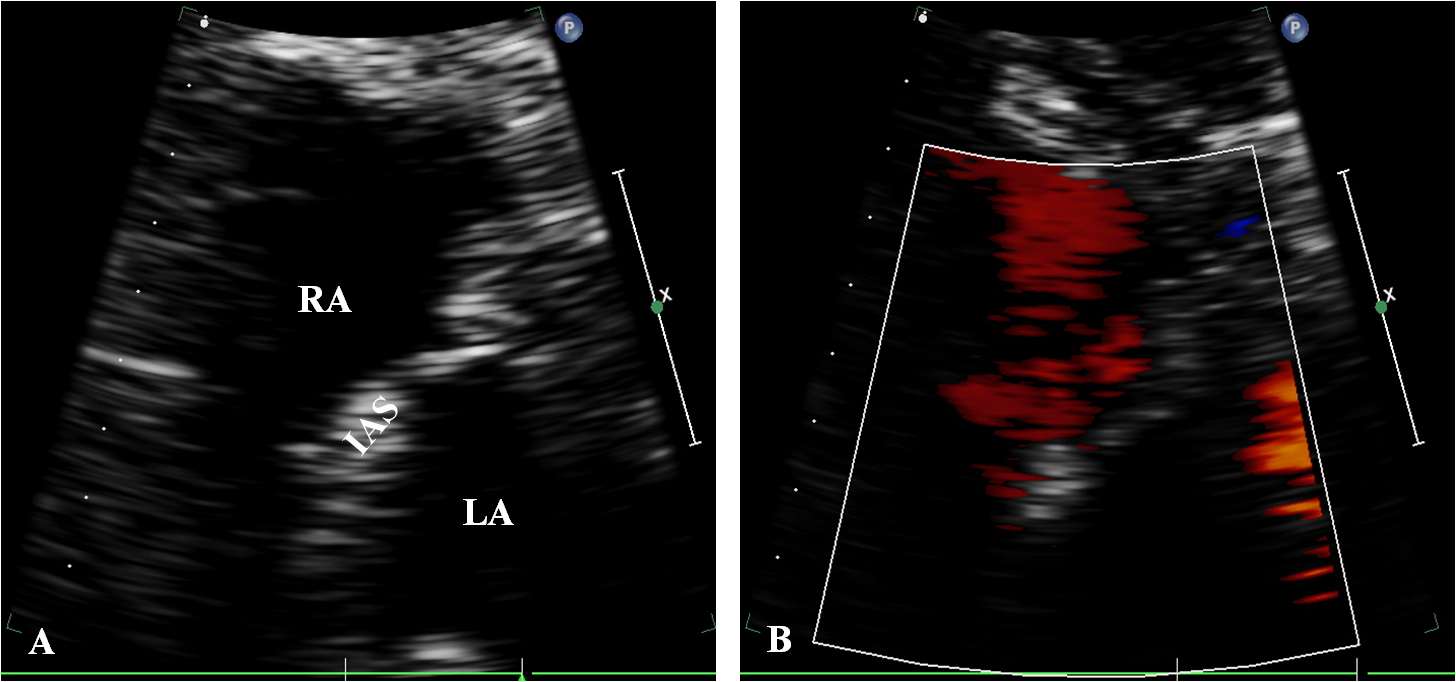


Supplemental table 1. Test of Protein S and Protein C

| No | Item | Result | Point | Unit | Reference range |
| --- | --- | --- | --- | --- | --- |
| 1. | Activity of protein S | PS:C | 83.9 | % | 76-135 |
| 2. | Activity of protein C | PC:C | 82.0 | % | 60-140 |

Supplemental table 2. Test of Tumor markers

| No | Item | Result | Unit | Reference |
| --- | --- | --- | --- | --- |
| 1. | AFP | 2.04 | ng/ml | <8 |
| 2. | CEA | 2.60 | ng/ml | <3.4 |
| 3. | CA19-9 | 14.80 | U/ml | <22 |
| 4. | CA-125 | 16.47 | U/ml | <35 |
| 5. | T-PSA | 0.839 | ng/ml | <3 |
| 6. | CYFRA21-1 | 1.89 | ng/ml | <3 |

AFP: alpha-fetal protein; CEA: carcinoembryonic antigen; CA19-9: carbohydrate antigen 19-9; CA-125: carbohydrate antigen 125; T-PSA: total prostate specific antigen; CYFRA21-1: cytokeratin 19 fragment.

Supplemental table 3. Test of Autoimmune factors

| No | Item | Result | Unit | Reference |
| --- | --- | --- | --- | --- |
| 1. | IGG | 11.90 | g/L | 8.00-15.50 |
| 2. | IGA | 2860.00 | mg/L | 836-2900 |
| 3. | IGM | 1010.00 | mg/L | 700-2200 |
| 4. | IGE | 106.32 | IU/ml | 0.1-150.0 |
| 5. | C3 | 1.4400 | g/L | 0.785-1.520 |
| 6. | C4 | 0.4610 | ↑ g/L | 0.145-0.360 |
| 7. | RF | <20.00 | IU/ml | <20.0 |
| 8. | CIC | 0.09 | O.D | <0.15 |
| 9. | ANA | Suspicious (±) |  | Negative (－) |
| 10. | DNA | Negative (－) |  | Negative (－) |
| 11. | RNP | Negative (－) |  | Negative (－) |
| 12. | SM | Negative (－) |  | Negative (－) |
| 13. | SSA | Negative (－) |  | Negative (－) |
| 14. | SSB | Negative (－) |  | Negative (－) |
| 15. | SCL | Negative (－) |  | Negative (－) |
| 16. | JO | Negative (－) |  | Negative (－) |
| 17. | RIB | Negative (－) |  | Negative (－) |
| 18. | PFB | 547.00 | ↑ mg/L | 190-500 |

ANA: antinuclear antibody; C3: complement C3; C4: complement C4; RF: rheumatoid factor; CIC: circulating Immune Complexes; DNA: anti-double stain DNA antibody; IGG: immunoglobulin G; IGA: immunoglobulin A; IGM: immunoglobulin M; IGE : immunoglobulin E; JO: anti-Jo-1 antibody; PFB: properdin factor B; RNP: anti-ribonucleo protein antibody; RIB: anti-Rib antibody ;SM: anti-SM antibody; SSA: anti-SSA antibody; SSB: anti-SSB antibody; SCL: anti-SCL-70 antibody.
